# Radioterapia Estereotáxica para Tratamento de Taquicardia Ventricular Recorrente na Doença de Chagas: Relato do Primeiro Caso na América Latina

**DOI:** 10.36660/abc.20220614

**Published:** 2023-02-24

**Authors:** Mauricio I. Scanavacca, Cristiano F. Pisani, Bernardo Salvajoli, Rodrigo M. Kulchetscki, Marina P. Mayrink, João Victor Salvajoli, Roberto Kalil

**Affiliations:** 1 Hospital das Clínicas Faculdade de Medicina Universidade de São Paulo São Paulo SP Brasil Unidade de Arritmia, Instituto do Coração (InCor), Hospital das Clínicas da Faculdade de Medicina da Universidade de São Paulo, São Paulo, SP – Brasil; 2 Hospital das Clínicas Faculdade de Medicina Universidade de São Paulo São Paulo SP Brasil Serviço de Radioterapia, Instituto do Câncer (ICESP), Hospital das Clínicas da Faculdade de Medicina da Universidade de São Paulo, São Paulo, SP – Brasil

**Keywords:** Radioterapia, Taquicardia Ventricular, Doença de Chagas

A taquicardia ventricular sustentada (TVS) recorrente é uma condição comum em pacientes com cardiopatia chagásica crônica (CCC). A ablação por cateter está indicada nos casos em que tratamento medicamentoso falha, em particular quando há recorrências ou choques do cardioversor-desfibrilador automático (CDI) frequentes.^1 , 2^ Outras terapias têm emergido como opção para casos refratários; dentre elas, a radioterapia ablativa esterotáxica (RAE) tem mostrado resultados promissores nos pacientes com cardiopatia isquêmica e não isquêmica;^3 , 4^entretanto, não há relatos da aplicação desse tratamento em pacientes com CCC.

## Relato de caso

Paciente do sexo masculino, 53 anos, em acompanhamento no Instituto do Coração (InCor) do Hospital de Clínicas da Faculdade de Medicina da Universidade de São Paulo (HC-FMUSP) desde 2018 devido a CCC e TVS recorrente; além disso, teve um CDI dupla-câmara implantado em 2014. Apresentava fração de ejeção do ventrículo esquerdo (FEVE) de 42% ao ecocardiograma transtorácico (EcoTT), e na evolução foram identificadas terapias apropriadas do CDI a despeito do uso de amiodarona 400mg/dia, metoprolol 200mg/dia, enalapril 40mg/dia, espironolactona 25mg/dia, furosemida 40mg/dia.

No período de novembro de 2020 a junho a 2021, o paciente foi internado várias vezes devido a episódios de TVS revertidos pelo CDI apesar da reimpregnação por amiodarona, sendo submetido a três ablações por cateter, duas delas complicadas por hemopericárdio e hemoperitônio, com necessidade de correção cirúrgica. Apesar de alguma melhora inicial possivelmente atribuída às ablações sequenciais, recorreu em junho de 2021 com repetidas terapias apropriadas do CDI, sendo proposta sua inclusão em protocolo de pesquisa para ablação da TVS por RAE. Encontrava-se em classe funcional II/III da New York Heart Association (NYHA), com piora da FEVE (20%). Após consentimento livre e esclarecido, o paciente aceitou ser submetido à RAE.

**Figura 1:**
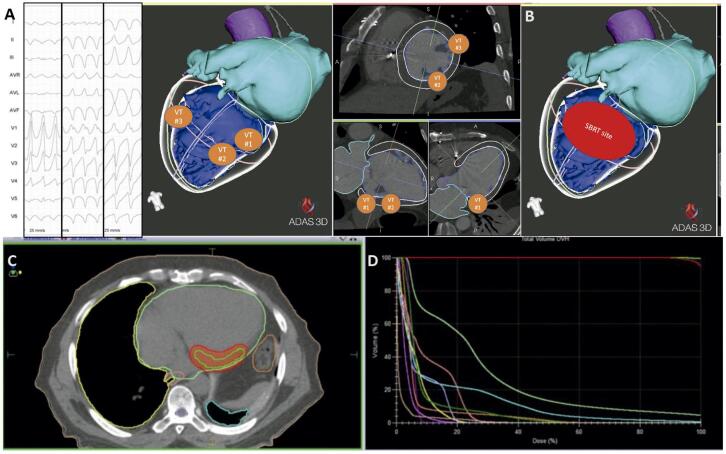
– Planejamento da radioterapia. Foram analisadas as morfologias das taquicardias ventriculares induzidas no estudo eletrofisiológico (A), e o local da saída foi marcado na reconstrução tridimensional da tomografia, uma vez os locais da saída foram marcados foi realizado o planejamento da área a ser irradiada (B). A área definida foi então manualmente marcada no sistema de radioterapia (C), que fez o cálculo das doses de tolerância (constraints) de cada estrutura vizinha (D).

A estratégia de planejamento da área a ser irradiada baseou-se em três informações: (1) morfologias das TVS e no mapa eletroanatômico dos procedimentos prévios, os quais demonstravam presença de cicatriz endo e epicárdica na região ínfero-látero-basal do VE com área de ativação lenta e canais de condução visualizados no *Ripple Mapping* , coerentes com a provável origem das TVS documentadas; (2) uma angiotomografia de coronária processada em *software* específico (ADAS3D^®^, Adas3D Medical SL, Barcelona, Espanha), que foi integrada com objetivo de facilitar a visualização da cicatriz e auxiliar na demarcação dos alvos da RAE ( Figura 1 ); e (3) um novo estudo eletrofisiológico, realizado com o intuito de avaliar as morfologias atuais de TVS, sendo induzidas três novas morfologias, com os locais de saída demarcados nas imagens do *software* ADAS 3D^®^.

Na sequência, uma tomografia quadridimencional foi realizada no setor de radioterapia, com o paciente na posição de tratamento e utilizando os acessórios de imobilização *blue bag* e *bodyfix* (BodyFIX^®^, Elekta, Stockholm, Suécia). Com esse conjunto de imagens, as equipes da radioterapia e da eletrofisiologia planejaram a área alvo a ser irradiada. Ao final do planejamento, foi realizado o contorno dos órgãos adjacentes para calcular as doses de tolerância de cada órgão. Após essas marcações, a equipe de física médica calculou a dose de radiação, a fim de proteger os órgãos vizinhos. O plano foi aprovado pelo radioterapeuta responsável e o tratamento realizado em 14/07/2021.

O tratamento foi realizado em sessão única que durou 15 minutos de irradiação e 30 minutos entre entrada e saída do paciente na unidade de radioterapia. A dose aplicada foi de 25 Gy, prescrito na isodose 80%. Os parâmetros de tratamento foram: *planning target volume* (PTV): 74,51 cc; *internal target volume* (ITV): 26,56; estômago Dmáx: 17 Gy / 5cc = 9,25Gy; esôfago Dmáx: 7,6Gy; Cólon Dmáx: 16,5Gy / 20cc = 8 Gy; medula Dmax: 5,3Gy; coração dose média: 6,9Gy. Durante o tratamento, o paciente permaneceu monitorizado, o CDI foi programado em modo assíncrono e com a detecção de taquiarritmias desligada.

No mesmo dia, após a radioterapia, o paciente apresentou evento de TVS tratado com *antitachycardia pacing* (ATP) e choque, e a avaliação eletrônica mostrou se tratar de evento semelhante aos anteriores. Apresentou náuseas de intensidade leve nos primeiros dias após o procedimento. O paciente permaneceu internado por mais dois dias, com exames laboratoriais e troponina seriada sem alterações significativas. O ecocardiograma também não revelou piora na função ventricular ou novas alterações segmentares.

**Figura 2:**
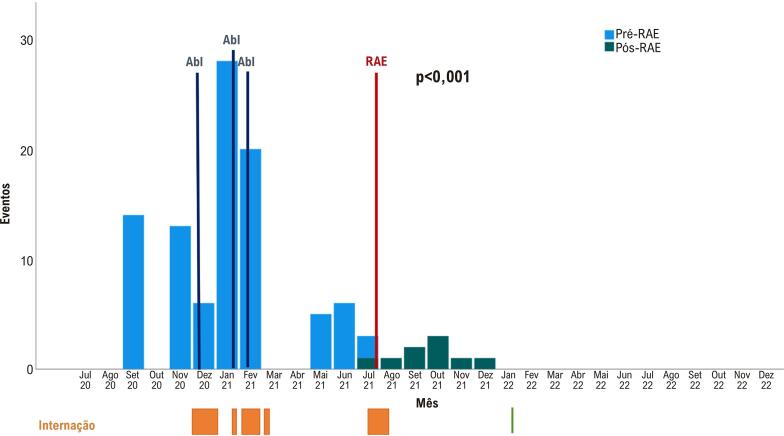
– Linha do tempo e gráfico de colunas com o número de terapias em cada mês (coluna azul antes e verde após radioterapia) e os momentos das ablações (linha vertical azul) e radioterapia (linha vertical vermelha). Em laranja estão os momentos de internação hospitalar por taquicardia ventricular, e em verde internação para troca de gerador do cardioversor-desfibrilador automático. Abl: ablação; RAE: radioterapia ablativa esterotáxica.

Durante o seguimento clínico, o paciente foi mantido com amiodarona 200mg e carvedilol 75mg por dia, além de enalapril, espironolactona e furosemida. Apresentou um episódio de TVS em agosto de 2021 revertido com ATP, dois em setembro, um deles revertido por choque do CDI, três em outubro, um em novembro e outro em dezembro, todos revertidos com ATP. Desde então não apresentou mais eventos de TV. Quando comparado aos 12 meses anteriores à radioterapia, foi observada redução significativa do número de episódios de TV (p <0,001) ( Figura 2 ).

Devido a quadro de hepatite aguda, a amiodarona foi suspensa temporariamente em novembro de 2021 e retornada em janeiro de 2022. O paciente apresentou lesão cutânea em pé direito com biópsia demonstrando melanoma acral lentiginoso, sendo encaminhado para acompanhamento com o serviço de oncologia. Em avaliação clínica em Julho de 2022, encontrava-se em classe funcional I da NYHA, com melhora da função ventricular (FEVE de 30%) ao EcoTT e sem palpitações ou síncopes. O Holter evidenciou redução do número de extrassístoles ventriculares de 68/h (13% do total de batimentos) no pré-tratamento para 3/h (< 1%) no pós. A última avaliação eletrônica do CDI, em Dez/22, não identificou arritmias nos 12 meses precedentes.

## Discussão

As ablações das TVS em pacientes com CCC são procedimentos complexos, na maioria das vezes necessitando de acesso epicárdico e com alta taxa de recorrência e morbidade.^2 , 5^A RAE tem crescido como uma nova opção terapêutica nos casos de TVS recorrente e cardiopatia estrutural. Sua efetividade e segurança foram inicialmente estudadas em pacientes com cardiopatia isquêmica e não isquêmica, não incluindo pacientes com CCC.^3 , 4 , 6^

Considerando a experiência já descrita nos pacientes com cardiopatia isquêmica e não isquêmica, acreditamos que a radioterapia poderia ser um procedimento alternativo também para o tratamento de pacientes com CCC e TVS refratárias. Optamos inicialmente pela realização do procedimento em um paciente para o qual a ablação por cateter já não era mais uma opção, por impossibilidade de acesso epicárdico e devido às complicações em dois procedimentos prévios. Uma das preocupações iniciais da radioterapia foi a proximidade de esôfago, estômago e cólon com a região lateral, posterior e basal do VE, frequente na TVS da CCC; porém, nesse paciente as doses de tolerância ( *constraints* ) foram adequadas para esses órgãos.

O planejamento da área a ser irradiada foi realizado em conjunto pelas equipes de eletrofisiologia e da radioterapia, sendo a decisão baseada nas morfologias de TVS induzidas em procedimentos prévios e em estudo eletrofisiológico realizado dias antes da radioterapia, além de exames de imagem com angiotomografia reconstruída em *software* específico e as imagens do mapeamento eletroanatômico com mapeamento de voltagem e mapeamento funcional buscando identificar os potenciais istmos das TVS, com irradiação dessa área buscando a modificação do substrato.

Após a radioterapia, o paciente apresentou recorrência de TVS nos primeiros 5 meses, sem apresentar novos episódios de TVS nos 12 meses seguintes. Esse achado decorre de o efeito da radioterapia na célula cardíaca não ser imediato, sendo observado inicialmente apoptose e inflamação e posteriormente ao redor do quinto ao sexto mês se estabelece a fibrose.^7^

Interessante que o paciente vinha apresentando piora progressiva da função ventricular e classe funcional, provavelmente associada às múltiplas recorrências de TVS e terapias do CDI, apresentou estabilização clínica e discreta melhora na fração de ejeção, inclusive com redução importante na densidade de extrassístoles ventriculares.

## Conclusão

Este é o primeiro caso reportado na literatura médica que sugere efeito benéfico da ERA no tratamento de um paciente com CCC e TVS refratária ao tratamento convencional. Entretanto, são necessárias informações a serem obtidas em casuísticas prospectivas com maior número de pacientes para estabelecimento dos riscos e benefícios desse procedimento em pacientes com essas características clínicas.
