# Supplementary material for: Radioterapia Estereotáxica para Tratamento de Taquicardia Ventricular Recorrente na Doença de Chagas: Relato do Primeiro Caso na América Latina
Source: Arq Bras Cardiol. 2023 Feb 24;120(2):e20220614. [Article in Portuguese] doi: 10.36660/abc.20220614 (PMC10263457; doi:10.36660/abc.20220614)
Supplement: Translation

# Translation: Stereotactic Body Radiation Therapy for Recurrent Ventricular Tachycardia in Chagas Disease: First Case in Latin America

**Authors:** Mauricio I. Scanavacca, Cristiano F. Pisani, Bernardo Salvajoli, Rodrigo M. Kulchetscki, Marina P. Mayrink, João Victor Salvajoli, Roberto Kalil

**Affiliations:** 1 Brazil Unidade de Arritmia, Instituto do Coração (InCor), Hospital das Clínicas da Faculdade de Medicina da Universidade de São Paulo, São Paulo, SP – Brazil; 2 Brazil Serviço de Radioterapia, Instituto do Câncer (ICESP), Hospital das Clínicas da Faculdade de Medicina da Universidade de São Paulo, São Paulo, SP – Brazil

**Keywords:** Radiotherapy, Ventricular Tachycardia, Chagas Disease

Recurrent sustained ventricular tachycardia (SVT) is a common condition in patients with chronic Chagas heart disease (CCHD). Catheter ablation is indicated when drug therapy fails, especially in cases of recurrences or frequent implantable cardioverter defibrillator (ICD) shocks.^1 , 2^Others therapies have emerged as an option for refractory cases; among them, Stereotactic body radiation therapy (SBRT) has shown promising results in patients with ischemic and non-ischemic heart disease;^3 , 4^however, there are no reports of application of this treatment in patients with CCHD.

## Case Report

A 53-year-old male patient has been followed up at Instituto do Coração (InCor) of Hospital das Clínicas da Faculdade de Medicina da Universidade de São Paulo (HC-FMUSP) since 2018 due to CCHD and recurrent SVT; moreover, he had a double chamber ICD implanted in 2014. He had a left ventricular ejection fraction (LVEF) of 42% on transthoracic echocardiography (TTE), and ICD-appropriate therapies were identified, despite the use of amiodarone 400 mg/day, metoprolol 200 mg/day, enalapril 40 mg/day, spironolactone 25 mg/day, and furosemide 40 mg/day.

From November 2020 to June 2021, the patient was admitted several times due to episodes of SVT reverted by ICD, despite amiodarone reimpregnation, and he underwent three catheter ablation attempts, two of them complicated by hemopericardium and hemoperitoneum, which required surgical correction. Although there was an initial improvement, possibly attributed to sequential ablations, the patient experienced a recurrence in June 2021, with repeated ICD-appropriate therapies, and his inclusion in research protocol for SVT ablation by SBRT. He was classified into New York Heart Association (NYHA) functional class II/III, with worsening of LVEF (20%). After providing his free informed consent, the patient accepted to undergo SBRT.

The strategy for planning the area to be irradiated was based on three findings: (1) morphology of SVTs and electroanatomic mapping of previous procedures, which show the presence of endo-epicardial scar at the inferior-lateral-basal segment of the LV with a slow area of activation and conduction channels visualized on Ripple Mapping, coherent with the probable origin of the documented SVTs; (2) a coronary angiography processed in a specific software (ADAS 3D^®^, ADAS 3D Medical SL, Barcelona, Spain), which was integrated in order to facilitate the scar visualization and help demarcate SBRT targets ( Figure 1 ); and (3) a new electrophysiological study, performed to assess the current SVT morphologies, in which three new morphologies were induced with exit sites demarcated in images from the ADAS 3D^®^software.

**Figure 1:** – Radiation therapy planning. Ventricular tachycardia morphologies induced in the electrophysiological study (A) were analyzed, and the exit site was demarcated on the three-dimensional reconstruction of computed tomography scans and, once exit sites were demarcated, there was the definition of the area to be irradiated (B). The defined area was then manually demarcated in the radiation therapy system (C), which calculated dose constraints for each surrounding structure (D).

Subsequently, a four-dimensional computed tomography was performed in the radiation therapy unit, with the patient in the treatment position and immobilized in a BlueBAG™ BodyFIX^®^vacuum cushion (Elekta, Stockholm, Sweden). With this set of images, the radiation therapy and electrophysiology teams planned the target area to be irradiated. At the end of the planning, adjacent organs were contoured to calculate dose constraints for each organ. After these delimitations, the medical physics team calculated the radiation dose in order to protect surrounding organs. The plan was approved by the radiation therapist in charge and the procedure was performed on 07/14/2021.

The treatment was performed in a single session lasting 15 minutes of irradiation and 30 minutes from patient’s arrival at the radiation therapy unit to his exit. The applied dose was 25 Gy, prescribed to the isodose 80%. Treatment parameters were: planning target volume (PTV): 74.51 cc, internal target volume (ITV): 26.56, stomach Dmax: 17 Gy/5cc = 9.25 Gy; esophagus Dmax: 7.6 Gy; colon Dmax: 16.5 Gy/20cc = 8 Gy; bone marrow Dmax: 5,3 Gy, mean heart dose: 6.9 Gy. During treatment, the patient continued to be monitored, and the ICD was programmed to the asynchronous mode and for not detecting tachyarrhythmias.

On the same day, after radiation therapy, the patient had a SVT event treated with ATP and shock, and electronic assessment showed that it was an event similar to the previous ones. He complained of mild nausea during the first days after the procedure. The patient remained hospitalized for two more days, and laboratory tests did not show significant changes, as well as serial troponin. Moreover, echocardiography did not reveal worsening of ventricular function or new segmental changes.

During clinical follow-up, the patient remained on amiodarone 200 mg and carvedilol 75 mg daily, in addition to enalapril, spironolactone, and furosemide. He had an episode of SVT in August 2021, which was reversed with antitachycardia pacing (ATP), two episodes in September, one of them reversed by ICD shock, three episodes in October, one in November, and another in December all of them reversed with ATP. Since then, the patient has not presented any VT event. In comparison to the 12 months prior to radiation therapy, there was a significant reduction in the number of VT episodes (p < 0.001) ( Figure 2 ).

**Figure 2:** – Time line and column graph showing the number of therapies in each month (blue column before radiation therapy, green column after) and times of ablations (blue vertical line) and radiation therapy (red vertical line). Times of hospitalization due to ventricular tachycardia are shown in orange, and times of hospitalization for changing the implantable cardioverter defibrillator generator are shown in green. Abl: ablation; SBRT: stereotactic body radiation therapy.

Due to the onset of acute hepatitis, amiodarone was temporarily suspended in November 2021 and resumed in January 2022. The patient presented with a skin lesion on the right foot, whose biopsy revealed acral lentiginous melanoma, and he was referred to the oncology service. In a clinical assessment in July 2022, the patient was classified into NYHA functional class I and showed improvement in ventricular function (LVEF of 30%) on the TTE and did not present with palpitations or syncope. The Holter results showed a decrease in the number of ventricular extrasystoles from 68/h (13% of total heart beats) before treatment to 3/h (< 1%) after treatment. The last electronic evaluation of the ICD, in Dec/22, did not identify arrhythmias in the previous 12 months.

## Discussion

SVT ablations in patients with CCHD are complex procedures, often requiring epicardial access and having high recurrence and morbidity rates.^2 , 5^SBRT has grown as a new therapeutic option in cases of recurrent SVT and structural heart disease. Its effectiveness and safety were initially studied in patients with ischemic and non-ischemic heart disease, not including patients with CCHD.^3 , 4 , 6^

Considering the experience already described in patients with ischemic and non-ischemic heart disease, we believe that radiation therapy could be an alternative procedure also for the treatment of patients with refractory CCHD and SVT. We initially decided to perform this procedure in a patient for whom catheter ablation was no longer an option, due to impossibility of achieving epicardial access and due to the complications that occurred in the two previous procedures. One of the initial concerns of radiation therapy was the proximity of esophagus, stomach, and colon with the lateral, posterior and basal segment of the LV, which is frequent in SVT resulting from CCC; however, this patient showed adequate dose constraints for these organs.

The planning of the area to be irradiated was jointly conducted by the electrophysiology and radiation therapy teams, and the decision was based on SVT morphologies induced in previous procedures and on a electrophysiological study performed a few days before radiation therapy, as well as on angiography scans reconstructed in a specific software and electroanatomic mapping images with voltage mapping and functional mapping in order to identify the potential isthmus of SVTs, with irradiation of this area in order to modify the substrate.

After radiation therapy, the patient experienced a recurrence of SVT in the first 5 months, without presenting new episodes of SVT in the following 12 months. This finding results from the fact that the effect of radiation therapy on heart cells is not immediate, initially occurring apoptosis and inflammation and subsequently, about the 5^th^to the 6^th^month, fibrosis.^7^

Interestingly, the patient, who had been presenting progressive worsening of ventricular function and functional class, probably associated with multiple recurrences of SVT and ICD therapies, experienced clinical stabilization and a slight improvement in ejection fraction, including a significant reduction in the density of ventricular extrasystoles.

## Conclusion

This is the first case reported in the medical literature to suggest the beneficial effect of SBRT in the treatment of a patient with CCHD and SVT refractory to conventional treatment. However, it is necessary to obtain information from prospective samples with a greater number of patients to define the risks and benefits of this procedure in patients with these clinical characteristics.
